# Science as a Teacher of Prophylaxis

**Published:** 1902-02

**Authors:** Samuel A. Hopkins

**Affiliations:** Boston, Mass.


### SCIENCE AS A TEACHER OF PROPHYLAXIS.[1]

BY SAMUEL A. HOPKINS, M.D., D.D.S., BOSTON, MASS.

Mr. President and Members of the Institute of Stomatology,—I am obliged to ask your indulgence for a few minutes while I review briefly a paper which I had the honor of reading before the Massachusetts Dental Society, at its annual meeting, held in Boston early last June.

The paper which I am about to read is a continuation of the one read at that meeting; and as that one has not yet appeared in print, and as it is necessary to the understanding of this paper that you should have some idea of the other, I will give you as briefly as possible a rough outline of some of the statements it contained.

I called attention to the remarkable mechanical and manipulative skill which our profession had attained, and expressed the belief that operative treatment could go no farther. Without decrying the immense good to humanity that such skill had wrought, I hinted that, with such remarkable facility for replacing lost teeth and repairing diseased ones at our command, both patient and dentist were taking chances with the natural organs that they would never dare take if no mechanical substitutes

---

[1] Second paper. Read before The New York Institute of Stomatology, October 1, 1901. Copyright, 1902, by Samuel A. Hopkins, M.D., DD.S., Boston, Mass.

were known. If our work of restoration were less successful, our efforts to prevent the loss of teeth would be more vigorous and emphatic.

I suggested that, in carrying on a warfare against dental caries, we must begin with the child at a very early age. In our own minds we must place the highest possible value upon the natural teeth, free ourselves from all thought of mechanical substitutes, and fight the battle as if the loss of a tooth were irreparable.

I spoke of Miller's theory as a practical working basis upon which to build up a system of treatment for carious teeth, and I drew attention to the theory of gelatin plaques as explaining some of the more obscure workings of Miller's theory. I showed that acid-producing bacteria, which we have come to accept as the destructive agents in the first stages of caries, do not receive their nutrition from the teeth themselves, but chiefly from particles of starchy and saccharine food found in the mouth.

I quoted the results of experiments which I had made to show that saliva varied as a culture medium, and I drew attention to the fact that bacteria would multiply much more rapidly in the thick, ropy saliva which we sometimes observe, than in the clear, watery fluid which we have come to look upon as the normal secretion of a healthy mouth.

I pointed out what I believed to be a marked association between this thick viscid saliva and rapidly progressing caries; but I regret to say that I was unable to state with any degree of certainty what caused the physiological change which gave rise to this condition of viscidity. I went on to state that so-called gelatin plaques were found more frequently and were more widely distributed in mouths containing this thick, mucus-laden saliva than in mouths in which the secretions were normal.

I stated that in producing decay artificially in the laboratory the greatest possible differences existed in the resisting power of the teeth used in such experiments. From this fact, as well as from the clinical experience of many careful observers, I was forced to conclude that living teeth differ materially in their susceptibility to and immunity from the action of caries. I also stated that caries was more easily produced by mixed cultures than by a pure culture of any of the lactic-acid-producing bacteria.

This, together with a tribute to Dr. D. D. Smith, of Philadelphia, for the courageous enforcement of his convictions in the prophylactic treatment of teeth, was the substance of the paper of which this is a continuation.

I wish now to call attention to other causes which tend to further the deterioration and destruction of human teeth. I shall point out some of the evil consequences of this retrograde metamorphosis, and shall offer a few suggestions as to the best means of working our way up to a better condition of the organs of mastication.

Whatever theories we may invent to explain the causes of dental caries, one fact stands out clearly and cannot be controverted,—namely, that caries is practically unknown among uncivilized races, and that degeneration of tooth-structure follows civilization like a Nemesis. Sometimes the changes which follow contact with civilization are so marked and so rapid that they seem almost like an epidemic. More frequently, however, the change is slow, working gradually, but insidiously and surely through long generations, until we wonder if this is, indeed, a process of evolution against which it is useless to strive. We must not forget, however, that evolution in the human race differs from that in the lower animals in this, that it is within the power of our intelligence to control it. Thought is supreme, and if we want good teeth, and want them badly enough, good teeth will be evolved to supply our needs and our desires. It is for us to say whether the human race shall become an edentulous race, or whether it is worth while to combat the degeneracy already begun and swing the huge pendulum the other way by eradicating disease and improving the structure of the human teeth.

This degeneracy is, as you well know, largely brought about by a loss of functional activity due largely to our civilized methods of preparing food. We resent with indignation food that necessitates vigorous mastication, and we allow our children to wash down their meals with copious draughts of water. Nature resents such treatment, and refuses to supply the necessary pabulum for maintaining a neglected organ. To functional activity she responds at once. When an organ is vigorously used waste material is taken up and active nutrition takes the place of stagnation. You might just as well confine an arm in a plaster-of-Paris cast, and expect it to retain its strength, as to deprive the teeth of the exercise of mastication and expect them to continue strong and well.

It is probable that the intensity of our modern life—the fearful nerve and brain tension that we are almost constantly under—has much to do with the degeneracy of our teeth. We note clinically a marked effect on the structure of teeth following an illness involving the nervous system, and we are familiar with the transparent, blue, rapidly decaying teeth of the highly wrought, intense nervous child. This burning up of the vital forces by excessive nervous and mental strain must be accepted as a factor in accounting for the deterioration of our teeth.

It is not improbable that the immediate cause of caries, which we believe to be acid-producing bacteria, is more active to-day than formerly, because of the too bountiful supply of starchy and saccharine food which enters the mouth. This food serves as a medium for bacterial activity. It is pointed out by Röse, as a result of experiments carried on in Baden and Thuringia, that the amount of calcium taken in through the food and water supply must be recognized as a factor in explaining the liability of teeth to carious action. This is probably true also as applied to the prenatal development of teeth. Another important factor in bringing about caries is so well recognized that I must apologize for mentioning it. I refer to irregularities. Irregularities may be due to the narrowing of the modern jaw, to the mixing of racial types, to faulty eruption of the permanent teeth, to adenoid growths, thumb-sucking, mouth-breathing, or to other causes. Whatever throws a tooth out of line and produces a lack of symmetry in the beautiful curve of the dental arch makes it difficult to properly cleanse the teeth. Whatever produces a faulty articulation makes it well-nigh impossible to keep up proper functional activity. A tooth without an antagonist is bound to deteriorate.

Besides these causes there are others which may be looked upon as accidental or at least as occasional, such as congenital imperfections, prolonged illness, pregnancy, chemical agents, and traumatism, but time will hardly permit of a consideration of these causes. There is, however, one other consideration that we must refer to in this connection. Dr. Michaels, of Paris, in his work on Sialo-Semeiology, says, "The saliva of adolescence contains a dextrinic principle (glycogen) susceptible of fermentation under the influence of ptyalin in the presence of earthy salts. In this way is obtained the dissolution of the earthy salts by the substitution of lactic acid for carbonic acid." If this statement means anything, it means that lactic acid is formed in the mouth without the aid of bacteria, and that lactic acid so formed is capable of attacking the teeth and destroying them. Since reading Dr. Michael's article I have sterilized and examined the saliva of a large number of children, and have in no instance been able to discover a trace of lactic acid that was not to be ascribed to acid-producing bacteria. While I have long believed that certain conditions of the saliva promoted carious action, I am equally sure that this is due to the fact that under certain conditions the saliva becomes a better culture medium and thus favors the increase of acid-producing bacteria. If it is true, as Dr. Michaels asserts, that lactic acid can be produced in the mouths of young people without the aid of bacteria, it is probably true also that the cases in which this change takes place are too infrequent to have any especial bearing on the subject we are now considering.

Having touched briefly upon the causes which assist in bringing about degeneration and loss of teeth, and before taking up the evil effects produced by diseases of these organs, let us pause a moment to consider how wide spread the evil really is.

Dr. Denison Pedley, of England, made some interesting studies of the teeth of school children in that country, and found that seventy-five per cent. of the children examined had diseased teeth. This is a low estimate as compared with the condition on the continent of Europe and in this country. Statistics indicate that nearly ninety-seven per cent. of all our public school children have carious teeth. It is not too much to say that thirty per cent. of all the teeth of school children between the ages of five and fifteen in the public schools of this country are diseased. This is appalling when we know that only a small proportion of these children will ever receive treatment at the hands of a competent dentist; and when we realize that without such treatment they must inevitably go from bad to worse, the situation becomes truly alarming. It is rendered more striking when we realize that in spite of unhygienic surroundings only two and a half per cent. of the Eskimos have carious teeth; while the Indian and Malay tribes, having been smirched, but not yet conquered, by civilization, still dwell in comparative ignorance of caries, only ten per cent. of their number being as yet afflicted.

It is not necessary to dwell on these facts to show what a menace to health and to mental and moral progress we have confronting us. Many of these unfortunate school children suffer with intermittent toothache for years; and thanking God for the intermissions, they bravely accept the condition as a part of their lives. What sort of mental and moral development can go on when opposed by such a handicap; and what an effect upon the child's health and physical development must such a condition bring about! I tell you, gentlemen, that physical and moral degeneracy, idleness, and crime may have a more intimate association with caries than we are quite willing to admit. To emphasize the necessity of a reformation in our care of the teeth, let us consider for a few minutes the relation of diseases of the dental organs to general diseases.

Three years ago Dr. Charles Stedman Bull, speaking before this society, pointed out the connection between diseases of the eye and dental lesions, in a paper so able that it attracted the attention of the medical profession throughout the country. Without now going into details, you may remember that he proved conclusively that keratitis, glaucoma, muscular paralysis, asthenopia, amblyopia without visible lesions, supraorbital neuralgia, and exophthalmos with and without cellulitis are frequently caused directly or indirectly by carious teeth. He showed that eye-complications of dental disease are of varied nature and may reach to the most superficial structures of the eye. He spoke most positively when he said that when we come to consider the lesions of the cornea and sclera, the cases reported in connection with diseased teeth are almost numberless. Cases of loss of accommodation from paralysis of the ciliary muscles have been shown to be in many instances due to diseased teeth, and optic neuritis ending in atrophy of the nerve and blindness has also been traced to carious teeth. This excellent paper of Dr. Bull's was printed in the INTERNATIONAL DENTAL JOURNAL of March, 1898, and will well repay careful perusal.

We are perfectly familiar with diseases of the nasal and accessory cavities which have their origin in dental lesions. We have too often witnessed antrum and aural disturbances dependent upon carious teeth not to fully appreciate the intimate relationship between these parts. It is a well-established fact that otalgia of a very pronounced type not infrequently arises from a carious tooth. Nervous interference with the nutrition of the middle ear may be due to dental disorders, and may cause impairment of hearing. In fact, when we consider the reflex disturbances due to diseases of the teeth we are at a loss for space to enumerate them.

When it comes to diseases of bacterial origin, we find that with few exceptions all bacteria find their way into the general system through the mouth. I have myself found the tubercle bacillus, the Klebs-Löffler bacillus, actinomycosis, and many pyogenic forms in mouths of supposedly healthy people. A correspondent of *La Revue Médicale* for May, 1896, reported one hundred and thirteen cases of lymphadenoma in children. In forty-one per cent. dental caries was the only cause that could be found, and it is fair to infer that in a much larger percentage complications of caries were present. Chronic glandular swellings in the neck are dependent upon caries in a majority of instances. It has been found in several cases of tuberculous infection that the bacillus invaded the organism through a decayed tooth. Many cases are reported to show that primary tuberculosis of the mouth, which happily is not very common, generally shows itself first around a diseased tooth or root. General infections of septicæmia followed by death are frequently reported, and in far too many cases these have their origin in a diseased tooth. Numerous cases of pyæmia, periostitis, ostitis, and metastatic abscesses resulting in death have been shown to have originated in carious teeth. When we come to consider gastric troubles, the number proceeding from imperfect mastication caused by a neglected or diseased condition of the mouth and teeth far exceeds those from all other causes put together.

I could go on multiplying indefinitely these cases in which general disease is dependent upon dental lesions, but I have, I think, said enough to show that our professional work can no longer be confined to its present narrow limits, but that we must face broader and more important questions than now occupy our minds, if we are to make a permanent and useful impression upon the human race.

The question immediately arises, Is it worth while? We are doing our work in a comfortable way, showing more or less skill, and, it cannot be denied, doing more or less good; and we have a comfortable income. Why excite ourselves about the rest of the world? Well, of course, if we are content to eat, drink, marry, and die, it is not worth while; but thank God that is not the kind of men the dental profession is composed of to any great extent! As I said in my first paper, it is one of the few professions in which men are striving to cut off the sources of their income by substituting prevention for cure. It is a profession that has had from its beginning the welfare of the patient at heart and has striven earnestly for the improvement of the human race. Scamps and charlatans belong to all trades and professions, but the proportion is comparatively small in the dental profession. The work is too hard and too exacting, and the dishonest and loose-fibred man can do better in some other walk of life. Therefore I feel that it is only necessary to show that this work of preventing caries is feasible, that it is important to the improvement of the human race, and that a fair degree of success awaits our efforts to have every honest member of the profession do his part in bringing about the desired reformation.

When I make the assertion that the development of the dental organs and the strength of the same is and will always be in ratio to their use; when I assert that lack of use will infallibly tend to weaken and to the suppression of these organs, I am simply stating a well-known scientific principle, and do not wish to be misunderstood. I do not know what the intention of the Almighty is in regard to the future of the human race, and I do not believe any one else knows. If we conscientiously believe that in the process of evolution the teeth are doomed; that it is fruitless to waste our time in combating the degeneracy that has already obtained so strong a foothold, it is our duty to help on the process by wholesale extraction, so that we may at least have clean gums and clean mouths and lessen thereby the danger of that general infection which I have shown to be associated with diseased teeth.

The thought is repulsive to you, but it is not illogical. As a matter of fact, strong teeth are associated with and are essential to the best types of manhood. If my statement seems startling I think I can show good grounds for making it. In the first place it has been shown by the examination of the teeth of school children in England, on the continent of Europe, and in this country that there does exist a ratio between the physical soundness and mental acuteness of the child and the condition of his teeth. I am not willing to pervert facts by saying that this ratio is very pronounced, but I firmly believe that it will be more emphatically shown when the investigations have been carried farther. The child with strong teeth will be found to have decidedly the best of it, both physically and mentally, and that he will be morally stronger will follow as a matter of course. As the age of the child advances the difference seems to be more marked, and when we reach the age when in this country boys enter college, the fact begins to be established in a remarkable degree.

It has been my fortune to have had for patients a very large number of college students, and my interest in college sports has brought me into intimate relations with the trainers, coaches, and captains of crews and teams. In this way I have had opportunity to examine the mouths of a large number of the members of "Varsity" crews and foot-ball teams, and of other college athletes. I have naturally been led to make inquiries of other dentists regarding this class of men, and have also gained a great deal of information from trainers and others interested in college athletics. I have not been able to classify and tabulate the results of my examinations, for in most cases such an examination would be hasty and casual,—usually made in the dressing-room before or after an afternoon's practice,—but the results are striking. The proportion of strong sets of teeth among these young men is greatly above the average, and can be seen in the most superficial examination.

I cannot deny that great intellectuality is often seen in association with weak teeth, nor can it be denied that men of large physique frequently have poor teeth, but that combination of vital energy and mental strength which goes to make commanders and vigorous pioneers is rarely found in association with frail teeth. Our beloved Washington was one of the rare exceptions. If it is true, then, that deterioration of the dental organs will cause a general deterioration in the human race, our duty is plain, and we are bound to apply our energies to combating this great evil. In my last paper,[1] which you can read at your leisure if the subject interests you, I marked out a line of treatment which will, I am persuaded, prevent a large percentage of decay and will carry a child through life without a large filling or other serious operation. Without repeating myself, it is sufficient to say that the plan consists in frequent examinations and frequent polishings. Once or twice in our professional lives we meet with a conscientious mother who insists upon having her child's teeth taken care of. If there is a speck or shadow on the infant teeth, in she comes and demands an appointment. She pesters us with questions as to the care of the child's teeth, and insists upon our making the most minute

[1] Dental Cosmos, October, 1901.

examinations and doing the most careful polishing. She makes life a burden to us by her insistance, and we are almost ashamed to make a charge for the slight and seemingly unnecessary operations which we perform. But what is the result? If a spot is discovered which marks the first step of caries, we polish and polish until it disappears, and no cavity results. The teeth strengthen, and we find that when the child grows up the teeth are strong and only a few small fillings have ever been inserted. We give ourselves small credit for this result, and in our blindness believe that the child's teeth were naturally strong and resistant to decay, when, as a matter of fact, our own efforts, called forth by one of those God-given mothers, have produced this wonderful result. Did you ever know any other result in such a case? Is it not almost inevitable that such a child will have excellent teeth? It is only necessary to apply this same treatment to all children to have uniformly strong and healthy teeth.

We ought to take the initiative, and not wait to be prodded into doing our duty. It is for us to educate the mother about the care of the mouth. We should point out the importance of proper mastication and show how suicidal it is to allow the little ones to wash down each mouthful with water. It is within our province to inquire into the feeding of the child and to make suggestions on this subject, and I can assure you that in most cases these suggestions will be received with gratitude and the attempt will be made to improve the faulty diet. I have very little faith in the practice of feeding special foods for a special purpose, because we know so little of the marvellous chemistry of digestion and assimilation that we cannot follow the food to its ulterior depository, and the phosphates that we feed to nourish the brain may, for aught we know, find their final resting-place in the joint of a big toe. We do know, however, that certain foods are insufficient to nourish certain organs; that finely bolted flour, for instance, will not build up tooth-structure, while whole wheat contains the nutrient ingredients.

With the knowledge we have of the means of prevention, with the readiness with which our views are accepted by our patients, there should be little or no trouble in reducing caries to a minimum in private practice. Three-fifths of the dental operations performed in the last twenty years were preventable. Will it be possible to make that statement at the end of the next twenty years, or shall we have succeeded in reducing that proportion? I have not spoken of brushing the teeth nor of the use of washes, for I have nothing to say that you do not already understand. There is, however, another important aspect to this question which we must consider, and that is the condition of the teeth of the poorer classes.

Poor teeth cause poor digestion. Irritability of the stomach produces a craving for alcohol, and alcohol causes crime. Ergo, unhealthy teeth lead to unhealthy morals. This may sound like the Darwinian theorem which demonstrates that the old maids of England are the cause of her vigorous manhood. The bone and muscle of Englishmen is derived from the excellent quality of the English beef and mutton. These fine cattle and sheep feed chiefly upon clover. The clover is fecundated by bees that carry the pollen from one plant to the other. Field-mice are the enemies of bees and destroy their nests. Cats are the enemies of field-mice and destroy them, and the old maids care for the cats and encourage their increase, and hence are the cause of the strength of the men. It is an interesting chain, but no such roundabout method of reasoning is needed to show that the proper conservation of the teeth is essential to health and happiness. So far as the well-to-do classes are concerned, I believe that the tide has already turned, and that the effect of prophylaxis is beginning to be felt, but the question of what we shall do for the teeth of the poor calls for our most thoughtful and prayerful consideration.

In the economy of human life the teeth are of no less importance than eyes, ears, throat, lungs, spine, or any other portion of our anatomy. But while for diseases of every other part of the body free hospitals and dispensaries offer to the poor every facility for treatment, there is not, to my knowledge, throughout the length and breadth of this land, a single place where a person unable to pay a fee can have the tortures of an aching tooth assuaged by competent hands. I do not forget the excellent work done in our college dispensaries, but that, as you know, is so small in amount compared to the great need of such work that it only points out more emphatically the need of free dispensaries for the poor. While we are trying to influence legislation, and are bringing this matter to the favorable notice of State and city governments, there should be little difficulty in getting a private endowment for one or two such institutions. There are ten or a dozen men before me to-night who could each raise fifty or a hundred thousand dollars among his own patients for such an object as this. These institutions, started perhaps by private means, would soon demonstrate that from economic motives their existence and usefulness should be maintained. The decrease of crime, the lessening of disease, the improvement in physical and mental conditions would be soon remarked, and these institutions would form an important part of our public education by teaching oral hygiene. That there would at first be opposition springing from the ignorance of the poor there is no doubt. They have an exaggerated dread of all dental operations. This would be soon overcome if the dispensaries were in the hands of wise and kindly men. Good teeth should be made a prerequisite for attendance in our public schools, just as vaccination is required at the present time. To bring about these changes we must gain the co-operation of medical men, and this we can do in a large measure through our personal acquaintance. We must influence the instructors in our dental schools so that this subject may occupy a more prominent place in the college curriculum. Dental sanitation should occupy a large space in our books on hygiene and in our dental and medical literature, and, most important of all, our dental societies should become interested and take the initial step in starting this important reformation. And that is what I am here for to-night. My convictions are from long and earnest study, and I have drawn no chimerical picture of the needs of our race; nor do I believe that if we give our hearts and our hands to the work of improvement, the undertaking will be found beyond our strength.

